# The effect of continuous ultrasound on chronic low back pain: protocol of a randomized controlled trial

**DOI:** 10.1186/1471-2474-12-59

**Published:** 2011-03-16

**Authors:** Safoora Ebadi, Noureddin Nakhostin Ansari, Nicholas Henschke, Soofia Naghdi, Maurits W van Tulder

**Affiliations:** 1 Rehabilitation Faculty, Tehran University of Medical Sciences, Iran; 2 The George Institute for Global Health, Sydney, Australia and EMGO Institute for Health & Care Research, Amsterdam, the Netherlands; 3 Professor of Health Technology Assessment, Department of Health Sciences and EMGO Institute for Health & Care Research, Faculty of Earth & Life Sciences, VU University Amsterdam, the Netherlands

## Abstract

**Background:**

Chronic non-specific low-back pain (LBP) is one of the most common and expensive musculoskeletal disorders in industrialized countries. Similar to other countries in the world, LBP is a common health and socioeconomic problem in Iran. One of the most widely used modalities in the field of physiotherapy for treating LBP is therapeutic ultrasound. Despite its common use, there is still inconclusive evidence to support its effectiveness in this group of patients. This randomised trial will evaluate the effectiveness of continuous ultrasound in addition to exercise therapy in patients with chronic LBP.

**Methods and design:**

A total of 46 patients, between the ages 18 and 65 years old who have had LBP for more than three months will be recruited from university hospitals. Participants will be randomized to receive continuous ultrasound plus exercise therapy or placebo ultrasound plus exercise therapy. These groups will be treated for 10 sessions during a period of 4 weeks. Primary outcome measures will be functional disability and pain intensity. Lumbar flexion and extension range of motion, as well as changes in electromyography muscle fatigue indices, will be measured as secondary outcomes. All outcome measures will be measured at baseline, after completion of the treatment sessions, and after one month.

**Discussion:**

The results of this trial will help to provide some evidence regarding the use of continuous ultrasound in chronic LBP patients. This should lead to a more evidence-based approach to clinical decision making regarding the use of ultrasound for LBP.

**Trial registration:**

Netherlands Trial Register (NTR): NTR2251

## Background

Low back pain (LBP) is the most frequent self-reported type of musculoskeletal pain, is often recurrent, and has important socio-economic consequences. Estimates of the prevalence of LBP and are as high as 33% for point prevalence, 65% for 1-year prevalence, and 84% for lifetime prevalence [1].

Similar to other countries in the world, LBP is a common health and socioeconomic problem in Iran. In a cross-sectional study in one of the largest car-manufacturing companies in Iran, the 1-year prevalence of self-reported LBP was 21% (20% males and 27% females). The prevalence rate of absence due to LBP was 5% per annum [2]. As part of a World Health Organization study, LBP was detected in 15.4% of the population under survey in Tehran (urban area) [3] and in 23.4% of the population in rural areas in Iran [4].

LBP is defined as pain and discomfort in the lumbosacral region, below the twelfth rib and above the gluteal crease. According to the recommended diagnostic triage, three types of back pain can be defined: 1) non-specific low back pain; 2) back pain with nerve root symptoms; and 3) back pain resulting from serious pathology (e.g. malignancy, fracture, ankylosing spondylitis, infection). Non-specific LBP, in which there is no recognized patho-anatomic cause, is usually a benign condition but without appropriate management can develop into chronic LBP. Using the traditional classification system, LBP is also categorized according to its duration from onset, as acute (<6 weeks), sub-acute (6 weeks - 12 weeks), and chronic (>12 weeks) [5,6]. Chronic LBP remains a common problem that many practitioners have to deal with in primary care, secondary care, and occupational health care.

The main objective of treatment for chronic LBP is for the patient to return to their desired level of activities and participation, as well as the prevention of chronic complaints and recurrences [7]. Many treatments are commonly used for LBP such as medication, physiotherapy, and surgery. Many of these interventions have been evaluated in randomized controlled trials and systematic reviews. Evidence shows that the effectiveness of some of the interventions is supported (e.g. exercise), while it shows that other interventions are not effective for LBP (e.g. laser therapy and traction) [8-10]. The number of randomized trials varies widely across interventions, from 61 for exercise therapy to 19 on back school and 4 on transcutaneous electrical nerve stimulation (TENS) [9,11,12].

Therapeutic ultrasound is used frequently in the treatment of LBP by many physiotherapists around the world and after more than 60 years of clinical use, there is still great controversy over its usefulness [13-16]. According to two recent clinical guidelines, the UK NICE guideline http://guidance.nice.org.uk/CG88 and the COST B13 European guideline for the management of LBP [5], there is no evidence regarding the benefit of using electrotherapy modalities such as interferential, laser, TENS, and ultrasound even though these modalities are commonly used in physiotherapy practice. The guidelines and recent systematic reviews [17] of therapeutic ultrasound have highlighted a need for further research to investigate the true effect of these modalities in the context of well conducted randomized controlled trials. As the application of ultrasound may have adverse effects for patients with LBP (e.g. because of the transmission of thermal energy), it is important to know whether the benefits outweigh the risks of this commonly used intervention.

This study will aim to investigate the effects of continuous ultrasound with pre-defined doses, in comparison with sham ultrasound, delivered in combination with a semi-supervised exercise program on pain intensity and function in patients with chronic LBP. This study has received ethical approval from the Tehran University of Medical Sciences.

## Methods

### Study design

A single blind randomized controlled trial in which participants with chronic LBP will be randomized to one of two treatment groups:

• Continuous ultrasound + semi-supervised exercise therapy

• Sham ultrasound + semi-supervised exercise therapy

### Study population

Patients aged between 18 and 65 years who present with chronic LBP will be recruited for this study.

### Inclusion and exclusion criteria

Patients with LBP who have pain for more than 3 months will be eligible. Patients with nerve root symptoms, underlying systematic or visceral disease, and specific conditions such as neoplasm, fractures, spondylolysthesis, spondylolysis, spinal stenosis, ankylosing spondylitis, previous low back surgery, and pregnancy will be excluded. The researcher will also ask potential participants if they take any medication for specific psychological problems in which case they will be excluded.

### Recruitment procedures

Recruitment will take place in two steps: firstly, orthopaedic surgeons from three university hospitals in Tehran, Iran will identify potentially eligible patients and refer them to the research centre at the Rehabilitation Faculty of Tehran University of Medical Sciences. Secondly, the researcher will conduct another screening for inclusion and exclusion criteria and make the final decision regarding eligibility. If eligible, the patient will be provided with oral and written information about the study. After having signed a consent form, patients will be randomized to one of the two treatment groups.

### Randomization

Opaque, sealed envelopes will be prepared to the total number of patients using a computer generated randomization schedule, with half of the envelopes being allocated to each group ensuring equal number of subjects in each group.

The list and the envelopes will be prepared by a statistician who is not involved with the study prior to commencement of the trial to ensure concealment of patient allocation.

### Sample size

According to the data of a randomised pilot study which involved 10 patients, the effect size based on the Functional Rating Scale (FRI) was calculated to be 1.83. Since the number of patients in the pilot study was few, we calculated the sample size for the current study using an effect size of 0.8. Considering the results of our previous article [18] and the results obtained from the pilot study we expect ultrasound to be more effective and have chosen a one-sided test to calculate the sample size. Assuming alpha and power to be 0.05 and 0.8, in a one-tailed test the total sample size needed is 42 (21 patients in each group). Accounting for 10% drop-out, the total number of patients required is 46 (23 patients in each group).

### Interventions

Subjects in each group will receive 10 sessions of treatment, each around 20 minutes, during a period of 4 weeks. All treatment, ultrasound delivery, and exercise prescription will be provided by the same licensed and experienced physiotherapist who will be instructed by the researcher about the study protocol.

### Ultrasound application

Therapeutic ultrasound is proposed to deliver energy to deep tissue sites through ultrasonic waves, to produce increases in tissue temperature or non-thermal physiologic changes [19]. Unlike ultrasound for medical imaging (which transmits ultrasonic waves and processes a returning echo to generate an image), therapeutic ultrasound is a one-way energy delivery which utilises a crystal sound head to transmit acoustic waves at 1 or 3 MHz and at amplitude densities between 0.1 and 3 W/cm^2 ^[16,19]. Ultrasonic energy causes soft tissue molecules to vibrate from exposure to the acoustic wave. This increased molecular motion generates frictional heat, thus increasing tissue temperature. Referred to as ultrasound's "thermal effects", this heating is proposed to increase collagen extensibility, increase nerve conduction velocity, alter local vascular perfusion, increase enzymatic activity, alter contractile activity of skeletal muscle, and increase nociceptive threshold [16,19].

While recent systematic reviews of therapeutic ultrasound have failed to identify a dose-response relationship [17], dosage ranges from 0.5 W/cm^2 ^to 3 W/cm^2 ^(spatial average temporal average) have been advocated to minimise adverse effects [20]. Recently published randomised controlled trials which have reported significant benefits of therapeutic ultrasound over placebo ultrasound have used dosages of 1 W/cm^2 ^to 1.5 W/cm^2 ^[18,21].

In the current study, the patients will receive continuous ultrasound using Enraf Nonius Sonoplus 434 operated at a frequency of 1 MHz and intensity of 1.5 W/cm^2 ^(spatial average temporal average). Slow circular movements will be applied by the transducer head over the paravertebral low back region. The duration of ultrasound application will be estimated for each patient using Grey's formula [22]:

For this study, the average local exposure time is planned to be one minute and the effective radiating area of the transducer head is 5 cm^2^. For a patient with an area of low back pain of 40 cm^2^, for example, the required total treatment time is: 1 min × (40 cm^2 ^/5 cm^2^) = 8 minutes.

### Placebo ultrasound

Patients in the placebo ultrasound group will receive the same duration of ultrasound with the apparatus switched on (so that patients will see the lights flashing on the machine) but without any current output. In this way, patients will be blinded for the ultrasound treatment. Success of this blinding, and consequently potential bias, will be evaluated by asking patients at the end of the intervention period if they think that they have received verum or placebo ultrasound.

### Exercise therapy

Exercise therapy is defined as "a series of specific movements with the aim of training or developing the body by a routine practice or as physical training to promote good physical health" [9]. Exercise therapy appears to be slightly effective for decreasing pain and improving function in adults with chronic LBP [9]. Studies on therapeutic exercises often fail to provide details on the specific exercise techniques used and the exact exercise protocol that was prescribed or followed (e.g. dose, timing, intensity) [23]. In our study both groups will receive pamphlets in which all the exercises that are going to be taught during the treatment period will be available in combination with pictures.

This semi-supervised exercise program will start with stretching routines for the lower limbs as well as the lower back. Then strengthening exercises for abdominal muscles and paravertebral muscles of the low back will be added to each session according to the physiotherapist's judgment of the patient's condition. Evidence suggest that lumbar extensor strengthening exercise administered alone or with co-interventions is more effective than no treatment and most passive modalities for improving pain, disability, and other patient-reported outcomes in chronic LBP [23]. The rationale behind choosing this exercise plan was because access to exercise facilities may be limited and patients can easily learn and practice the prescribed exercises at home.

Patients will be asked to perform the exercises on a daily basis (every day). To encourage aerobic activity, patients will be advised to stay active during the day and try to walk for at least 15 minutes before exercising, which can also act as a warm up. In each session the patient will be asked to perform the exercises in front of the therapist to check for correctness. The therapist will then decide whether the patient should be progressed by making the exercises more difficult or increasing the number of repetitions. After completion of treatment sessions patients will be advised to continue staying active and will visit the clinic once a week to check their exercises and continue progression until the follow-up measuring session.

### Avoiding co-interventions

Patients will be requested not to commence new pain medications during the intervention period (from the beginning of treatment sessions until the end of follow-up period) and not to participate in any other exercise or treatment program. Co-interventions, including use of regular pain medication, will be measured during follow-up to evaluate potential bias.

### Outcome measures

Patient's specifications such as age, sex, body mass index, and pain history will be recorded in a demographic questionnaire at the baseline assessment. All primary and secondary outcome measures will be measured by the researcher at baseline, the final treatment session (after 4 weeks), and at a follow-up measurement after 3 months.

Primary outcome measures will be

• Functional disability due to LBP measured by the Functional Rating Index.

• Pain measured on a visual analogue scale (VAS)

### Functional Rating Index

The Functional Rating Index (FRI) is an instrument specifically designed to quantitatively measure the subjective perception of function and pain in the spinal area in a clinical environment. It has shown to also have satisfactory reliability and validity [24,25]. The FRI is a 10-item questionnaire consisting of 5-point Likert scales. The patient ranks his or her perceived disability at the present time by selecting one of the five points of the scale (0 - "no pain or full ability to function"; 4 - "worst possible pain and/or unable to perform this function at all"). The total score is obtained by summing the item scores and expressed as percentage from zero (no disability) to 100% (severe disability).

### Visual analogue scale

A visual analogue scale (VAS) is a commonly used, quick, reliable, and valid means to measure pain intensity in a variety of clinical contexts. This scale is a 100-mm horizontal line, where 0 mm indicates ''No pain'' and 100 mm indicates ''Unbearable pain.''

Secondary outcome measures will be

• Paravertebral muscle fatigue during a Biering-Sorensen test using surface electromyography and analysing the changes in fatigue indices after the treatment.

• Lumbar flexion and extension range of motion using the modified-modified Schober test

Reduced endurance capacity of back extensor muscles has been associated with chronic LBP [26,27]. Electromyography (EMG) spectral indices and their shift towards lower frequencies have been validated as tools to objectively monitor local lumbar back extensor fatigue in both healthy and LBP populations [28,29]. In this study we use EMG indices to evaluate whether the addition of ultrasound to exercise therapy will result in improved endurance outcomes.

### Biering-Sorensen test

The Biering-Sorensen test is a method for evaluating paravertebral muscle fatigue which was first introduced by Hansen and then modified by Biering-Sorensen [26]. In this test the subject will be asked to keep an unsupported prone horizontal position as long as possible, while their legs and hips are fixed to a bed. We will use a two part hydraulic bed in which the section under the upper body can be brought down slowly by pressing on an electronic button. The examiner will then ask the patient to keep his/her horizontal position. During the test, EMG activity of iliocostalis lumborum, multifidus and gluteus maximus will be recorded bilaterally using an 8 channel surface EMG recorder (DATA Log Biometrics Ltd). Surface EMG activity of the dominant side biceps femoris will also be recorded. The eighth channel will be connected to a digital goniometer. The end point of holding time would be when the patient fails from the horizontal position by more than 5 degrees as recorded by a digital goniometer.

### Recording surface EMG activity

Usually, assessment of fatigability in the Biering-Sorensen test is based on measuring endurance time, but this is hampered by subjective qualities such as motivation and tolerance to discomfort or pain. Electromyographic (EMG) spectral index compression toward lower frequencies has been employed to overcome this problem by measuring objectively the back extensor fatigability in isometric endurance contractions [30]. After shaving and cleaning the skin, the following electrodes will be attached:

#### Iliocostalis lumborum and multifidus

The determination of muscle fibre orientation is based on the recommendations of De Foa et al [31].

#### Gluteus maximus

At the midpoint of a line running from the inferior lateral angle of the sacrum to the greater trochanter [32].

#### Biceps femoris

At the midpoint of a line running between the ischial tuberosity and the proximal head of the fibula [32].

The digital goniometer will be positioned on the lumbar region according to the manual instruction of the apparatus. Surface EMG of the isometric activity of these muscles will be recorded during holding time. Holding time, median and mean power frequency slopes and intercepts, as well as the slope of the standard deviation of lumbar angle will be extracted from raw EMG using MATLAB version 7 and analysed by SPSS, version 17.

### Lumbar range of motion

Lumbar flexion and extension range of motion will be measured according to the modified-modified Schober (MMS) method. The MMS test has shown to produce reliable measurements of spinal flexion and extension in patients with LBP (Pearson's r = 0.78 to 0.89 for flexion and 0.69 to 0.91 for extension) compared with use of the double inclinometer technique (Pearson's r = 0.13 to 0.87 for flexion and 0.28 to 0.66 for extension), with less time to obtain the measurement [33].

In this method the two posterior superior iliac spines (PSIS) will be connected with a line on the skin and the middle of the line (first mark) and 15 cm above (second mark) will be marked. As the patient moves into lumbar flexion or extension, the distance between the two marks is measured and subtracted from the 15 cm distance in the neutral position [33].

### Data collection and analysis

An intention-to-treat analysis will be performed. In the case of dropouts and withdrawals, the last recorded values for the main outcome measures will be used in the analysis. We will check if the two groups are similar for the most important demographic characteristics and all outcome measures at baseline. In case of finding any differences we will adjust for them in a regression analysis. Between-group differences in the change of scores of all outcome measures will be analysed using the Statistical Package for the Social Sciences (SPSS) version 17. We will consider whether the data meet parametric or non-parametric conditions. If parametric, ANOVA, independent and paired t-tests will be used and in case of non-parametric conditions we will use Kruskal-Wallis, Mann-Whitney and Wilcoxon signed-rank tests. All analyses will be performed blinded for treatment allocation.

## Discussion

The fact that there are more than 20 types of treatment for chronic LBP, each of which has multiple subcategories, is a testament that no single approach has yet been able to demonstrate its definitive superiority [34]. For example, exercise therapy is one promising treatment option, but there is still no consensus upon which kind is the most effective [9]. This situation makes it very challenging for clinicians, policy makers, insurers, and patients to make decisions regarding which treatment is the most appropriate for chronic LBP.

Despite the widespread use of therapeutic ultrasound as one of the most popular and commonly used modalities in the field of physiotherapy for LBP patients, there is still limited evidence of its effectiveness. Only a few RCTs have investigated the effect of ultrasound in treating patients with chronic LBP; often with varying methodology qualities and very small sample sizes, and have not been able to provide evidence regarding its usefulness.

The RCT we have described will evaluate the effectiveness of therapeutic ultrasound in addition to exercise therapy in patients with chronic LBP. The advantages of this study would be comparing the ultrasound with placebo ultrasound, which would clarify the value of adding ultrasound to a semi-supervised exercise program. Limited possibilities for double blinding can be a potential limitation to this study. Meanwhile we will evaluate potential bias by asking the patients whether they think they received real or sham ultrasound on the last treatment session. Recruiting a sufficient number of patients will be one of the most challenging elements of this study since it will be conducted without substantial funding.

## Competing interests

The authors declare that they have no competing interests.

## Authors' contributions

SE and NNA came up with the original concept for the study. NH, NS, and MvT helped to design the study and contributed to the development of the protocol. SE wrote the first draft of the protocol with help from the other authors. All authors read and approved the final manuscript.

## Pre-publication history

The pre-publication history for this paper can be accessed here:

http://www.biomedcentral.com/1471-2474/12/59/prepub
